# Identifying patients at high risk for carbapenem-resistant Enterobacterales (CRE) carriage on admission to acute care hospitals: validating and expanding on a public health model

**DOI:** 10.1017/ice.2025.7

**Published:** 2025-04

**Authors:** Radhika Prakash-Asrani, Chris Bower, Chad Robichaux, Barney Chan, Jesse T. Jacob, Scott K. Fridkin, Jessica Howard-Anderson

**Affiliations:** 1 Emory University School of Medicine, Department of Medicine, Division of Infectious Diseases, Atlanta, GA, USA; 2 Emory University School of Medicine, Department of Medicine, Division of Biomedical Informatics, Atlanta, GA, USA; 3 Georgia Emerging Infections Program, Atlanta, GA, USA

**Keywords:** Carbapenem-Resistant Enterobacterales, Prediction Modeling, Surveillance

## Abstract

**Objective:**

Validate a public health model identifying patients at high risk for carbapenem-resistant Enterobacterales (CRE) on admission and evaluate performance across a healthcare network.

**Design:**

Retrospective case-control studies

**Participants:**

Adults hospitalized with a clinical CRE culture within 3 days of admission (cases) and those hospitalized without a CRE culture (controls).

**Methods:**

Using public health data from Atlanta, GA (1/1/2016–9/1/2019), we validated a CRE prediction model created in Chicago. We then closely replicated this model using clinical data from a healthcare network in Atlanta (1/1/2015–12/31/2021) (“Public Health Model”) and optimized performance by adding variables from the healthcare system (“Healthcare System Model”). We frequency-matched cases and controls based on year and facility. We evaluated model performance in validation datasets using area under the curve (AUC).

**Results:**

Using public health data, we matched 181 cases to 764,408 controls, and the Chicago model performed well (AUC 0.85). Using clinical data, we matched 91 cases to 384,013 controls. The Public Health Model included age, prior infection diagnosis, number of and mean length of stays in acute care hospitalizations (ACH) in the prior year. The final Healthcare System Model added Elixhauser score, antibiotic days of therapy in prior year, diabetes, admission to the intensive care unit in prior year and removed prior number of ACH. The AUC increased from 0.68 to 0.73.

**Conclusions:**

A CRE risk prediction model using prior healthcare exposures performed well in a geographically distinct area and in an academic healthcare network. Adding variables from healthcare networks improved model performance.

## Introduction

Infections due to carbapenem-resistant Enterobacterales (CRE) are difficult to treat and associated with significant morbidity and mortality.^1^ Patients can also be colonized with CRE which can lead to transmission of CRE to other patients, healthcare workers, and the environment.^2^ The healthcare environment including hospital sinks, countertops, and patient equipment can harbor CRE and increase the risk of healthcare facility outbreaks.^3–5^

To minimize the risk of healthcare-associated transmission, the US Centers for Disease Control and Prevention (CDC) recommends that healthcare facilities have strategies in place to identify patients with a history of CRE “carriage” (infection or colonization) on admission.^6^ Prompt identification allows for initiation of infection prevention measures including contact isolation and informs appropriate antibiotic selection if directed therapy is needed.^7,8^ However, rapid identification of patients with CRE carriage on admission requires obtaining perirectal cultures, which is labor-intensive, expensive, and potentially uncomfortable for patients. In a recent survey of clinicians involved in infection prevention through the Emerging Infections Network, only 22% reported that their primary facility performed routine active surveillance for CRE.^9^

Clinical risk prediction tools are increasingly used to aid in early detection of patients with multidrug-resistant organisms (MDROs).^10^ Risk factors for CRE carriage include prior healthcare exposures (including recent hospitalizations and intensive care unit [ICU] admissions), presence of indwelling medical devices, comorbidities including diabetes, poor functional status, and prior receipt of antibiotics.^10–14^ The CDC-funded Chicago Prevention and Intervention Epicenter previously created a CRE prediction model using a public health, statewide hospital discharge dataset to identify patients at high risk for CRE carriage on admission.^15^ This model performed well, however, performance of other models has been variable.^16^ It is unknown how well a public health model will perform when it is applied in a single academic healthcare network. Therefore, the objectives of our study, were to 1) externally validate the Chicago Epicenter model using public health surveillance data from Atlanta, GA and 2) evaluate the performance of a similar model using data from a single academic healthcare system and determine if including additional variables could improve the predictive model performance.

## Methods

### Study design, data sources

For both study objectives, because CRE culture is a rare outcome, we used a retrospective case-control study design to create a predictive model identifying patients at high risk for having a clinical CRE culture on admission.

#### External validation of the Chicago Epicenter Model

We used public health surveillance data from the CDC-funded Georgia Emerging Infections Program (EIP) to identify cases from 1/1/2016 to 9/1/2019. Georgia EIP performs active, population- and laboratory-based surveillance of CRE in the 8 counties of Georgia Health District 3 in metropolitan Atlanta, GA. CRE cases are identified by routine queries of laboratory automated testing instruments for carbapenem-resistant *Escherichia coli*, *Klebsiella pneumoniae*, *Klebsiella oxytoca*, *Klebsiella aerogenes*, and *Enterobacter cloacae* isolated from a sterile site or urine clinical culture.^17^ We identified controls from the Georgia Hospital Discharge dataset which is a public health dataset that includes administrative data for all acute care hospitals (ACH) and long-term acute care hospitals (LTACH) encounters in Georgia.

#### Evaluation of Healthcare System Model

We used electronic health record (EHR) data and identified cases and controls from 1/1/2015–12/31/2021 from four hospitals in a single academic healthcare network in Atlanta, Georgia. Hospital A is a 605-bed hybrid academic–community, tertiary-care hospital. Hospital B is a 751-bed academic quaternary-care hospital that performs solid organ and hematopoietic stem cell transplantation. Hospitals C and D are 410-bed and 167-bed community hospitals, respectively. In 2023, these hospitals had over 76,000 acute care admissions combined. None of these hospitals perform routine surveillance screening for CRE.

### Case and control definitions

For both study objectives, we defined cases as ACH encounters for adults (≥18 years) who had CRE identified from a clinical culture within the first three days of admission. Only the first qualifying encounter per patient was included. We defined CRE as growth of *E. coli*, *K. pneumoniae*, *K. oxytoca*, *K. aerogenes*, or *E. cloacae* resistant to meropenem, doripenem, or imipenem (MIC ≥ 4 µg/mL). For the external validation analysis, since EIP performs surveillance in all healthcare settings (not only ACHs) we also included individuals with CRE identified within 90 days prior to an ACH encounter as a case, assuming these patients would still be colonized with CRE on admission. For the external validation analysis, we only had data on CRE cultures from normally sterile sites or urine. For the evaluation of the healthcare system model, we included clinical cultures from all body sites.

Controls were defined as ACH encounters for adults during the same period who did not have CRE identified. We also excluded from controls any patient who had a CRE culture at any time during our study period. Patients with known CRE are usually empirically isolated on hospital admission and would not be representative controls. We frequency matched cases and controls based on year of admission and healthcare facility. This allowed us to control for potential confounding from temporal changes in CRE prevalence, variation in the geographic distribution and any facility-specific factors.

### Statistical analysis and model development

For each study objective, we first used descriptive statistics to characterize cases and controls based on demographics, comorbidities, and key prior healthcare exposure variables using unadjusted logistic regression. For each multivariable model, we split the data into training (80%) and validation (20%) datasets. We report adjusted odds ratios (OR) from the training datasets and explore the impact of each predictor variable by standardizing the model coefficients using a common scale and ranking them. We evaluated model goodness of fit using Akaike Information Criterion (AIC) in the training dataset and model performance using Area Under the Curve (AUC) in both the training and validation datasets.

#### External validation of the Chicago Epicenter Model

We replicated the Chicago Epicenter model using the available independent risk factors identified in their model including age, number of encounters in ACHs in the prior 365 days, mean length of stay in ACHs in the prior 365 days, number of encounters in LTACHs in prior 365 days, mean length of stay in LTACHs in the prior 365 days, and prior hospital admissions with an infection diagnosis in the prior 365 days. We used the Georgia Hospital Discharge dataset to identify the number and length of ACH and LTACH encounters within the state in the prior 365 days. Since our aim was to create a model for admissions solely to ACHs, we excluded the variable referring to current admission at an LTACH which was used in the original Chicago Epicenters model. To determine the prior infection diagnosis, we used a validated list of infection diagnosis codes (ICD-9 and ICD-10) that were previously demonstrated to be a surrogate for antibiotic exposure, provided to us by the Chicago Epicenters group.^15^ Variable selection techniques were not employed as we aimed to validate the original Chicago Epicenters model.

#### Evaluation of healthcare system model

We first created a “Public Health Model” which included the variables from the Chicago Epicenters model that were available to us using data from a single academic healthcare network. These included age, number of encounters in ACHs in the prior 365 days, mean length of stay in ACHs in the prior 365 days, and prior hospital admissions with an infection diagnosis in the prior 365 days. Variables related to prior ACH encounters only included data from encounters within the same academic healthcare network. We did not have data on prior LTACH admissions, so these variables were excluded.

Next, we created a “Healthcare System Model” to evaluate if adding other variables known to be risk factors for CRE would improve the model performance.^10–13,15^ Candidate variables included admission to an ICU in the prior 365 days, malignancy, Elixhauser score, total antibiotics days of therapy (DOT) in the prior 365 days, beta-lactam antibiotic DOT in the prior 365 days and diabetes. The weighted Elixhauser score was calculated using the “Comorbidity” package and “Elixhauser Score” option in R Studio. ICD-9 billing codes were used prior to 2016 and ICD-10 codes were used after this. Malignancy was defined as any patient meeting the metastatic cancer or solid tumor criteria of the Elixhauser score, and diabetes was defined as any patient meeting the uncomplicated or complicated diabetes definition in the Elixhauser score. The same list of infection diagnosis codes used in the validation analysis were used to determine the prior infection variable. The antibiotic DOT variables were log-transformed to account for skewness. All variables in the full model were checked for collinearity based on the variance inflation factor which was less than 3, indicating minimal multicollinearity. For variable selection, we used Best Subset logistic regression (“bestglm” package in R Studio) which is a technique that evaluates all possible combinations of predictor variables and identifies the best combination based on the AIC. The approach allows for assessing the relative importance of variables and their joint predictive power. ORs were estimated using penalized logistic regression to account for the rare outcome.

### IRB approval

The Emory University Institutional Review Board approved this study with a waiver of the patients’ informed consent as all data was collected retrospectively.

## Results

### External validation of the Chicago Epicenter Model

Using the Georgia EIP data, we identified 181 CRE cases that were frequency matched to 764,408 controls. Cases were more likely to be older and Black (Supplemental Table 1). Nearly two-thirds of cases had a prior infection diagnosis as compared to a quarter of controls (OR 5.9, 95% CI 4.3–8.0). Cases had an increased number of ACH (OR 1.1; 95% CI 1.07–1.12) and LTACH (OR 5.4, 95% CI 4.3–6.8) admissions in the prior year and longer mean lengths of stay (SD) in these respective facilities (11.4 [13.4] vs 2.4 [4.8] days in ACH and 5.7 [15.1] vs 0.2 [2.9] days for LTACH) compared to controls (Supplemental Table 1).

In the training dataset for the multivariable model, we included 145 cases and 611,527 controls. Nearly all the independent risk factors identified in the Chicago Epicenters model were also significantly associated with CRE in Georgia (Table 1). Prior infection diagnosis (adjusted OR [aOR] 3.6, 95% CI 2.5–5.3), age 65–79 years (aOR 2.5, 95% CI 1.6–3.9) and mean length of stay in prior ACH encounters (aOR 1.03, 95% CI 1.02–1.03) were the three most impactful variables in the model based on standardized ranking of coefficients. The model performed well with an AUC of 0.80 (95% CI 0.77–0.84) in the training dataset and an AUC of 0.85 (95% CI 0.79-0.91) in the validation dataset (Table 1).

**Table 1.** Validation of the Chicago Epicenter model predicting Carbapenem-Resistant Enterobacterales carriage using Georgia public health data

	aOR^a^	95% CI		Rank
Prior infection diagnosis^b^	3.6	2.5	5.3	1
Age: 65-79 vs 18–49 years	2.5	1.6	3.9	2
Mean LOS (days) in prior acute care hospitalizations^b^	1.03	1.02	1.03	3
Age: ≥ 80 vs 18–49 years	1.5	0.8	2.7	4
Prior no. acute care hospitalizations^b^	1.05	1.00	1.10	5
Age: 50–64 vs 18–49 Years	1.2	0.8	2.0	6
Prior no. LTACH admissions^b^	2.8	2.0	3.9	7
Mean LOS (days) in prior LTACH admissions^b^	1.0	1.0	1.0	8
*Training Model AIC*	2502.8			
*Training Model AUC (95% CI)*	0.80 (0.77, 0.84)			
*Validation Model AUC (95% CI)*	0.85 (0.79, 0.91)			

Abbreviations: Adjusted odds ratio (aOR); Akaike Information Criterion (AIC); Area Under the Curve (AUC); Confidence Interval (CI); Long-Term Acute Care Hospitalizations (LTACH); Length of Stay (LOS).

a Adjusted odds ratios and 95% confidence intervals were estimated using the training dataset (145 cases; 611,527 controls).

b In prior 365 calendar days.

### Evaluation of healthcare system model

Using data from a single academic healthcare network, we identified 91 cases that were frequency matched to 384,013 controls. Cases and control were similar in terms of race and sex (Table 2). In the unadjusted analysis, individuals requiring ICU admission in the prior year (OR 8.1, 95% CI 5.0–13.0) and those with a prior infection diagnosis (OR 8.1, 95% CI 5.4–12.2) were 8 times more likely to have a clinical CRE culture on admission. Other significant variables included age (OR 1.02, 95% CI 1.01-1.03), number of encounters in ACHs in the prior year (OR 1.1, 95% CI 1.1–1.2), mean length of stay in the prior ACH encounters (OR 1.03, 95% CI 1.02–1.04), malignancy (OR 3.0, 95% CI 1.7–5.4), Elixhauser score (OR 1.05, 95% CI 1.04–1.07), diabetes (OR 2.2, 95% CI 1.4–3.3), antibiotic DOT (OR 1.8, 95% CI 1.6–2.0) and beta-lactam antibiotic DOT (OR 2.2, 95% CI 1.9–2.5) (Table 2).

**Table 2.** Key characteristics and univariable associations using data from an academic healthcare network

	Cases (N = 91)	Controls (N = 384,013)	Unadjusted OR	95% CI	
Age, median (IQR)	67 (58-75)	60 (44-72)	1.02	1.01	1.03
Male sex	50 (55)	170,848 (45)	1.5	1.0	2.3
Race and ethnicity					
Non-Hispanic Black	35 (39)	145,779 (38)	1.2	0.7	1.9
Non-Hispanic White	33 (36)	160,953 (42)	*Ref*		
Other^a^	23 (25)	77,281 (20)	1.5	0.9	2.5
Prior no. acute care hospitalizations, median (IQR)^b^	1 (0-3)	0 (0-1)	1.1	1.1	1.2
Mean LOS (days) in acute care hospitalizations, mean (SD)^b^	10.5 (12.8)	2.5 (5.5)	1.03	1.02	1.04
Previous admission to ICU^b^	23 (25)	15,426 (4)	8.1	5.0	13.0
Malignancy	13 (14)	20,125 (5)	3.0	1.7	5.4
Prior infection diagnosis^b^	44 (48)	39,747 (10)	8.1	5.4	12.2
Diabetes	38 (42)	94,616 (25)	2.2	1.4	3.3
Previous antibiotic DOT, median (IQR)^b^	14 (0-38)	0 (0-0)	1.8	1.6	2.0
Previous beta-lactam DOT, median (IQR)^b^	1 (0-16)	0 (0-0)	2.2	1.9	2.5
Elixhauser score, median (IQR)	23 (11-34)	10 (0-20)	1.05	1.04	1.07

Abbreviations: Days of Therapy (DOT); Intensive Care Unit (ICU); Inter Quartile Range (IQR); Standard Deviation (SD).

Values are number (%) unless otherwise stated. Data includes all cases and controls.

a Other race and ethnicity includes: Hispanic (3%); Asian, Native Hawaiian or Other Pacific Islander, American Indian or Alaskan Native, Multiple Race (4%); and Unknowns (14%).

b In the prior 365 calendar days.

**Figure 1.**
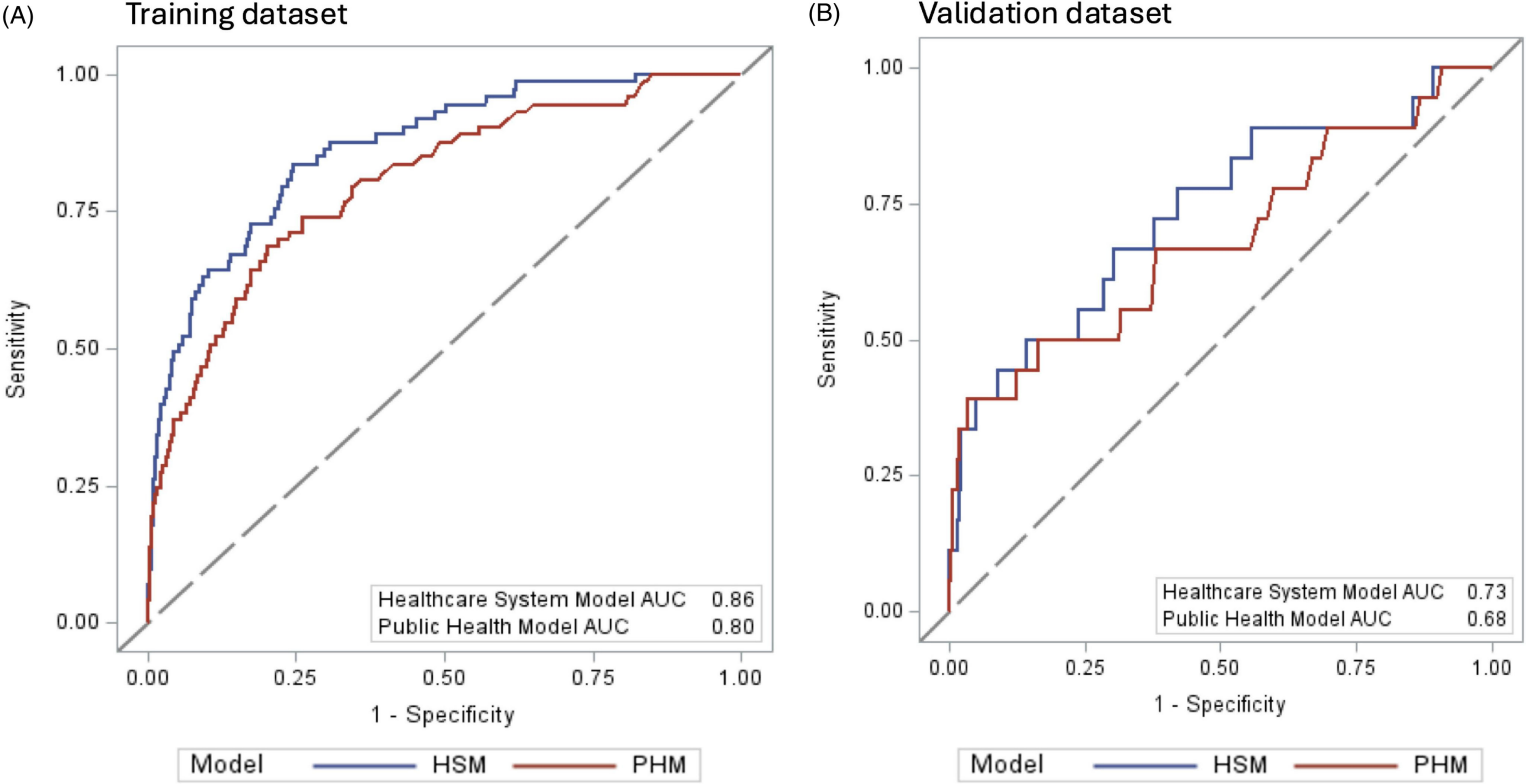
Receiver Operating Characteristic (ROC) Curves. The ROC curves illustrate the performance of the Healthcare System Model (HSM) (blue line) and Public Health Model (PHM) (red line). The x-axis represents the false positive rate (1-specificity), while the y-axis represents the true positive rate (sensitivity). Panel A displays the ROC curves using the training dataset and Panel B displays the ROC curves using the validation datasets. In Panel A, the area under the curve (AUC) for the HSM is 0.86 (95% CI 0.82–0.91), and the AUC for the PHM is 0.80 (95% CI 0.75–0.85). In Panel B, the AUC for the HSM is 0.73 (95% CI 0.60–0.87) and the AUC for the PHM is 0.68 (95%CI 0.53–0.83). A higher AUC indicates better overall model performance. *Abbreviations: HSM: Healthcare System Model; PHM: Public Health Model; AUC: Area Under the Curve*.

In the multivariable analysis, we included 73 cases and 307,211 controls in the training dataset. In the “Public Health Model,” designed to be similar to the Chicago Epicenter model, all variables remained independently associated with clinical CRE culture. The AUC for this model was 0.80 (95% CI 0.75–0.85) in the training dataset and 0.68 (95% CI 0.53–0.83) in the validation dataset (Table 3 and Figure 1).

**Table 3.** Comparison of multivariable models predicting carbapenem-resistant Enterobacterales carriage using data from an academic healthcare network

	Public Health Model				Healthcare System Model			
	aOR^a^	95% CI		Rank	aOR^a^	95% CI		Rank
Prior infection diagnosis^b^	6.3	4.0	10.1	1	3.0	1.8	5.0	3
Age	1.02	1.01	1.03	2	1.01	1.00	1.03	5
Prior no. acute care hospitalizations^b^	1.2	1.1	1.3	3				
Mean LOS (days) in acute care hospitalizations^b^	1.02	1.01	1.03	4	1.01	1.01	1.03	7
Previous admission to ICU^b^					2.4	1.4	4.1	6
Elixhauser score					1.04	1.02	1.06	1
Previous antibiotic DOT^b^					1.4	1.2	1.7	2
Diabetes					1.8	1.2	2.9	4
*Training Model AIC*	1240.3				1161.8			
*Training Model AUC (95% CI)*	0.80 (0.75, 0.85)				0.86 (0.82, 0.91)			
*Validation Model AUC (95% CI)*	0.68 (0.53, 0.83)				0.73 (0.60, 0.87)			

Abbreviations: Adjusted odds ratio (aOR); Akaike Information Criterion (AIC); Area Under the Curve (AUC); Confidence Interval (CI); Length of Stay (LOS); Days of Therapy (DOT); Intensive Care Unit (ICU).

a Adjusted odds ratios and 95% confidence intervals were estimated using the training dataset (73 cases and 307,211 controls).

b In prior 365 calendar days.

To improve the Public Health Model, we created the Healthcare System Model where additional variables were eligible for model inclusion. The final most parsimonious model for predicting clinical CRE culture on admission included the following variables, ranked by order of importance: Elixhauser score, antibiotic DOT in prior year, prior infection diagnosis, diabetes, age, admission to the ICU in the prior year, and mean length of stay in ACH encounters in the prior year (Table 3). The AUC of this model was 0.86 (95% CI 0.82–0.91) using the training dataset and 0.73 (95% CI 0.60–0.87) using the validation dataset (Table 3, Figure 1).

## Discussion

Here we demonstrated that a model developed using public health data from Chicago, IL performed reasonably well at identifying patients at high risk for having a clinical CRE culture on admission when applied to public health data from a geographically distinct area and to clinical data from a single academic healthcare network. Applying the model to an academic healthcare network and adding commonly available variables in the EHR improved the model performance. All covariates included in the final multivariable model (Elixhauser score, antibiotic DOT in the prior year, infection in the prior year, diabetes, age, ICU admission in the prior year, and mean length of stay in ACH encounters in the prior year) were independently associated with clinical CRE culture. The original Chicago Epicenter model focused on capturing patients’ prior healthcare exposures, particularly those related to infection and antibiotic use. These variables were also found to be associated with CRE when the model was applied to Georgia. This is consistent with prior literature demonstrating healthcare exposures are important predictors of CRE infection.^10–14,16^ In the final Healthcare System Model, mean length of stay in ACH encounters and prior diagnosis of infection were variables originally included in the Chicago model that were retained. However, the most impactful variable in the model became the Elixhauser score, a marker of individual comorbidity burden. In this final model, we did not have data on prior LTACH encounters, and data on prior ACH encounters was limited to those in the same healthcare network. These differences may help explain the change in the relative importance of the predictor variables and why the number of prior ACH encounters was dropped from the final Healthcare System Model. One hypothesis is that data on prior ACH and LTACH admissions captures a similar risk for CRE as the Elixhauser score, representing a patient’s medical complexity.

Identifying which patients have CRE carriage on admission is an important public health initiative but remains challenging for hospitals to implement. Our work demonstrates that a model originally developed by the Chicago Epicenter is broadly applicable. Tailoring the model to an individual academic healthcare network using additional data improved model performance by an increase in AUC of 0.05. However, whether this increase is clinically meaningful is unknown. Even hospitals with limited data analytic capabilities could start using the original public health model to identify patients at high risk for CRE. To replicate our study, healthcare systems would still ideally need to have access to a dataset of CRE cases to determine the model coefficients. Future work is needed to standardize model coefficients.

As a practical next step, we have integrated the Healthcare System Model into our EHR. We are prospectively evaluating this model by running it on all new admissions and testing patients determined to be at high risk for CRE. In the future, hospitals could determine their own model thresholds that would trigger additional actions such as screening for CRE and/or empiric contact isolation. These thresholds may vary based on the community prevalence of CRE and the resources or preferences of healthcare systems. Ideally, this proactive approach would limit the transmission of CRE within a hospital or healthcare network and decrease healthcare-associated outbreaks of CRE. Prior work has shown that predictive models used to identify and screen patients at high risk for CRE can decrease the incidence of CRE colonization but have the most impact on public health when also combined with a statewide registry identifying patients with known CRE.^18^

This study’s strengths include that we used multiple datasets, including one that includes population-based surveillance of CRE, to externally validate a previously created model predicting patients with clinical CRE cultures. Our study also has limitations. First, clinical CRE culture is a rare outcome, and so our datasets had substantially more controls than cases. The large number of controls could have led to many covariates being identified as statistically significant and potential overestimation of model fit statistics, including AUC. This may explain why the AUC in the external validation of the Chicago model increased in the validation dataset and the wide confidence intervals observed in the AUC values. Second, while ideally we would identify all patients with CRE carriage (infection and colonization), we only had data from clinical and not surveillance cultures to determine who had CRE on admission. We therefore could have misclassified some patients with asymptomatic carriage of CRE as controls. Third, the evaluation of the Healthcare System model only included data on prior ACH encounters from the same healthcare network and we do not have data on how often patients move between healthcare networks in this region. Lastly, our definition of CRE did not include patients with isolates only resistant to ertapenem, which differs from the current CDC surveillance definition, though is more specific for patients with carbapenemase-producing CRE, the most concerning CRE.^19,20^

In conclusion, we demonstrated that prediction models incorporating variables related to prior healthcare exposures and individual comorbidity burden can identify patients at high risk for CRE and are likely broadly applicable across the U.S. These models can be used to help healthcare facilities identify patients that may warrant surveillance testing for CRE. Future work is needed to determine the best strategies for real-time implementation of the model and to prospectively evaluate model performance when used in combination with active surveillance testing. The use of predictive modeling to inform surveillance testing could also be applied to other MDROs that are public health threats, including *Candida auris* or carbapenem-resistant *Acinetobacter baumannii*.

## Supporting information

Prakash-Asrani et al. supplementary material Prakash-Asrani et al. supplementary material

## Supplementary material

The supplementary material for this article can be found at https://doi.org/10.1017/ice.2025.7
