# Supplementary material for: Identifying patients at high risk for carbapenem-resistant Enterobacterales (CRE) carriage on admission to acute care hospitals: validating and expanding on a public health model
Source: Infect Control Hosp Epidemiol. 2025 Feb 14;46(4):398–403. doi: 10.1017/ice.2025.7 (PMC12015620; doi:10.1017/ice.2025.7)
Supplement: Prakash-Asrani et al. supplementary material [file S0899823X25000078sup001.docx]

**Supplemental Table 1. Key Characteristics and Univariable Associations using Georgia Public Health Data**

|  | **Cases**  **(N = 181)** | **Controls**  **(N = 764,408)** | **Unadjusted OR** | **95% CI** | |
| --- | --- | --- | --- | --- | --- |
| Age |  |  |  |  |  |
| 18-49 years | 36 (20) | 304,337 (40) | *Ref* |  |  |
| 50-64 years | 39 (22) | 186,430 (24) | 1.8 | 1.1 | 2.8 |
| 65-79 years | 76 (42) | 185,511(24) | 3.5 | 2.3 | 5.1 |
| ≥80 years | 30 (17) | 88,130(12) | 2.9 | 1.8 | 4.7 |
| Male sex | 78 (43) | 309,936(41) | 1.1 | 0.8 | 1.5 |
| Race |  |  |  |  |  |
| Black | 101 (56) | 346,799 (45) | 1.5 | 1.1 | 2.0 |
| Other^a^ | 10 (6) | 65,339 (9) | 0.8 | 0.4 | 1.5 |
| White | 70 (39) | 352,270 (46) | *Ref* |  |  |
| Prior infection diagnosis^b^ | 119 (66) | 187,768 (25) | 5.9 | 4.3 | 8.0 |
| Prior no. acute care hospitalizations, median (IQR)^b^ | 2 (1–4) | 0 (0-1) | 1.1 | 1.1 | 1.1 |
| Mean LOS (days) in acute care hospitalizations, mean (SD)^b^ | 11.4 (13.4) | 2.4 (4.8) | 1.0 | 1.0 | 1.0 |
| Prior no. LTACH admissions, median (range)^b^ | 0 (0–3) | 0 (0–6) | 5.4 | 4.3 | 6.8 |
| Mean LOS (days) in LTACH admissions, mean (SD)^b^ | 5.7 (15.1) | 0.2 (2.9) | 1.0 | 1.0 | 1.0 |

Values are number (%) unless otherwise stated

*^a^Other Race includes American Indian or Alaska, Native Hawaiian, Asian, Multiracial, Other*

*^b^In the prior 365 calendar days*

*Abbreviations: Long-Term Acute Care Hospitalizations (LTACH); Length of Stay (LOS); Inter Quartile Range (IQR); Standard Deviation (SD)*
